# Targeted Deletion of HIF-1α Gene in T Cells Prevents their Inhibition in Hypoxic Inflamed Tissues and Improves Septic Mice Survival

**DOI:** 10.1371/journal.pone.0000853

**Published:** 2007-09-05

**Authors:** Manfred Thiel, Charles C. Caldwell, Simone Kreth, Satoshi Kuboki, P. Chen, Patrick Smith, Akio Ohta, Alex B. Lentsch, Dmitry Lukashev, Michail V. Sitkovsky

**Affiliations:** 1 Laboratory of Immunology, National Institute of Allergy and Infectious Diseases, National Institutes of Health, Bethesda, Maryland, United States of America; 2 Department of Anaesthesiology, Klinikum Grosshadern, University of Munich, Munich, Germany; 3 Trauma, Sepsis and Inflammation Research Group, Department of Surgery, College of Medicine, University of Cincinnati, Cincinnati, Ohio, United States of America; 4 Department of Research, Shriner's Hospital for Children, Cincinnati, Ohio, United States of America; 5 New England Inflammation and Tissue Protection Institute, Northeastern University, Boston, Massachusetts, United States of America; Centre de Recherche Public-Santé, Luxembourg

## Abstract

**Background:**

Sepsis patients may die either from an overwhelming systemic immune response and/or from an immunoparalysis-associated lack of anti-bacterial immune defence. We hypothesized that bacterial superantigen-activated T cells may be prevented from contribution into anti-bacterial response due to the inhibition of their effector functions by the hypoxia inducible transcription factor (HIF-1α) in inflamed and hypoxic areas.

**Methodology/Principal Findings:**

Using the Cre-lox-P-system we generated mice with a T–cell targeted deletion of the HIF-1α gene and analysed them in an *in vivo* model of bacterial sepsis. We show that deletion of the HIF-1α gene leads to higher levels of pro-inflammatory cytokines, stronger anti-bacterial effects and much better survival of mice. These effects can be at least partially explained by significantly increased NF-κB activation in TCR activated HIF-1 α deficient T cells.

**Conclusions/Significance:**

T cells can be recruited to powerfully contribute to anti-bacterial response if they are relieved from inhibition by HIF-1α in inflamed and hypoxic areas. Our experiments uncovered the before unappreciated reserve of anti-bacterial capacity of T cells and suggest novel therapeutic anti-pathogen strategies based on targeted deletion or inhibition of HIF-1 α in T cells.

## Introduction

The surprisingly low incidence of serious complications after normal immune response to pathogens suggests the functioning of regulatory mechanisms that limit the collateral inflammatory damage to normal cells. It was suggested that it is the damage to the microvasculature and the ensuing decrease in oxygen supply (local tissue hypoxia) that may serve as primary signals of excessive tissue damage and need to de-activate immune cells [1]. One of the hypoxia-generated signals of excessive inflammation could be the accumulation of extracellular adenosine [2], while the second signal could be provided by a hypoxia-sensing molecule [1]. It was proposed that the overactive T-cells in inflamed and hypoxic areas are down-regulated by A_2A_ adenosine receptors [1] and hypoxia inducible factor 1α (HIF-1α) [3], [4] which may act in concert [5], [6]. While the genetic evidence for the critical role of A_2A_ adenosine receptors in down-regulation of activated immune cells *in vivo* has been provided using A_2A_ receptor deficient mice [2], [6], thereby confirming the overall validity of this “anti-inflammatory hypoxia” hypotheses, the anti-inflammatory role of HIF-1α in T cells *in vivo* remained to be proven using appropriate genetic models. The hypothesis of HIF-1α being *anti-inflammatory* in T cells seems to be counter-intuitive, since the myeloid cell-targeted deletion of HIF-1α resulted in the *loss* of inflammatory response by myeloid cells leading to the impression that HIF-1α in myeloid cells is *pro-inflammatory* [7]. Despite these findings, we reasoned that T cells may be different from macrophages and neutrophils, which are known to be much more dependent on glycolysis-derived ATP and therefore on glycolysis-controlling HIF-1α- [7]. We considered that since HIF-1α is not as critical for activated T cells' short term survival as it is for myeloid cells, it may play an important T cell-down regulating role in inflamed and hypoxic tissue microenvironments *in vivo* by acting in concert with other hypoxia-triggered mechanisms [1], [5]. To directly test this we used mice with T cell targeted deletion of HIF-1α gene. The present studies of bacterial sepsis show that T–cell specific deletion of HIF-1 α in mice results in: i) higher levels of pro-inflammatory cytokines; ii) stronger anti-bacterial effects of T cells and granulocytes and iii) better survival of mice from cecal ligation and puncture (CLP)-induced bacterial sepsis [8]. *In vitro* studies to clarify the possible mechanism of the *in vivo* observations revealed much faster cell proliferation and enhanced activation of NF-κB in HIF-1α deficient T cells. Quantitative real-time RT-PCR showed higher NF-κB p50 subunit expression in TCR stimulated T cells upon HIF-1α deletion, suggesting that the HIF-1α-mediated pathway is at least in part responsible for the suppression of NF-κB dependent cell proliferation and pro-inflammatory cytokine production. These experiments uncover the before unappreciated reserve of pro-inflammatory capacity of T cells *in vivo* and suggest novel therapeutic strategies when there is a need to enhance activity of T cells in hypoxic and/or inflamed tissues. We propose to inhibit HIF-1α in order to relieve from inhibition the “anti-bacterial” T cells during immune response to drug-resistant bacteria.

## Results and Discussion

### T-cells are present in hypoxic inflamed peritoneum but do not seem to contribute to anti-bacterial immune response

The comparison of survival rates of WT (n = 10) vs. T cell deficient RAG-2 knockout (n = 10) mice revealed no differences suggesting that T cells do not contribute to an anti-sepsis immune response in our model of sepsis (data not shown). We tested whether this could be explained by i ) the lack of T cells' presence in hypoxic inflamed areas or ii) by hypoxic inhibition of activated T cells in the local inflamed microenvironment.

**Figure 1:**
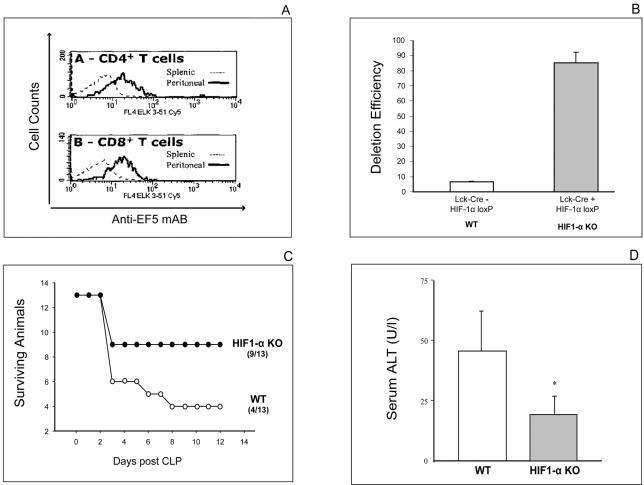
Increased survival and decreased bacterial sepsis-associated tissue damage of mice with T-cell targeted deletion of HIF-1α. A: *Use of the hypoxic marker EF5 reveals that CD4+ and CD8+ T cells have been exposed to low oxygen tension (hypoxic) conditions in the peritoneum during sepsis in mice.* Single cell suspensions from peritoneal lavage fluid and spleens were analyzed by flow cytometry using anti-EF5 mAb (Elk3-52 Cy5). B: *High Efficiency of Cre recombinase-mediated deletion of HIF-1* α *in T-Cells.* Efficiency of deletion was calculated by quantitative real-time PCR as described. Constitutively synthesized HIF-1 α mRNA was detected in control (lck-Cre negative) but not in (lck-Cre positive) HIF1 α gene targeted mice. N = 3 per group. C: *T cell lineage specific HIF-1* α *deficient mice are more resistant to lethal sepsis after cecal ligation and puncture procedure.* Mice underwent CLP and were observed for mortality. N = 13 per group. p = 0.0326, Logrank (Mantel-Cox). D: *T cell lineage specific HIF-1* α *deficient mice have less sepsis-associated liver damage as evaluated by levels of ALT transaminase activity in serum.* Serum samples obtained from mice 72 hrs after CLP. *:p<0.05 vs. WT, N = 5–6 per group.

The use of the *in vivo* hypoxic marker EF5 [9] allowed to discount the possibility of T cells avoiding hypoxic areas in order to maintain the highest levels of activation [10]. The labelling of both CD4+ and CD8+ peritoneal T cells *in vivo* by EF5 is diagnostic of their exposure to a less than 1% oxygen tension [9] in inflamed areas of the peritoneum (**Fig. 1A** **).**

To test whether the failure to observe T cells' contribution in anti-bacterial response and pathogen destruction was due to their negative regulation by hypoxia-stabilized and TCR-activation induced HIF-1α, we studied newly developed mice with T cell targeted deletion of the HIF-1 α gene. We hypothesized that if HIF-1α was indeed one of the mediators of immunosuppressive effects of hypoxia [10] by inhibiting T cells, then it would be expected that HIF-1α gene-deficient T cells would be resistant to inhibition in inflamed and hypoxic areas of peritoneum. This, in turn, would be expected to result in uninhibited production of e.g. IFN-γ and of other pathogen-destroying cytokines by activated T cells, as well as in the overall enhancement of the anti-bacterial host response and better survival.

### Selective disruption of HIF-1 α in T cells rescues mice from septic death and decreases bacterial burden-associated tissue damage

The possible contribution of T cells may have been overlooked in models of sepsis with early (<24 hour) lethal outcomes, since T cells may require longer than 24 hrs to be recruited to and activated in inflamed environment. Therefore, we have adapted a more long-lasting model of sepsis with ∼50% mortality after 72 hours of CLP [8] to allow sufficient time for T cell recruitment and activation in inflamed peritoneum so that T cells will have an opportunity to contribute to the anti-bacterial response. Comparative studies of mice with the confirmed HIF-1 α deletion in their T cells (**Fig. 1B**) supported the view that HIF-1α in T cells did inhibit them during sepsis. This was reflected in a statistically significant increase in survival of mice with HIF-1α-deficient T cells as compared with their HIF-1 α expressing littermates (**Fig. 1C**). Survival between lck-Cre (+) and lck-Cre (−) control mice was not different, demonstrating that the difference in survival between lck-Cre (+) HIF-1 α loxP and lck-Cre (−) HIF-1 α loxP mice was indeed due to Cre mediated HIF-1 α knock-down (data not shown). In another control, the sham CLP surgery did not result in any mouse mortality (data not shown).

The significantly higher survival rates of mice with T cell-targeted deletion of HIF-1 α gene (n = 27) as compared with control HIF-1 α expressing mice (n = 29) were also observed in other independent experiments in different mice facilities (p = 0.040, log rank test; C. Caldwell, data not shown).

The HIF-1 α deficient mice also had much less sepsis-associated tissue damage in different organs as demonstrated by an e.g. significant decrease in levels of liver enzymes (**Fig. 1D**). Histological analysis of livers revealed less apoptotic hepatocytes 72 hrs after CLP in HIF-1 α deficient mice (not shown).

**Figure 2:**
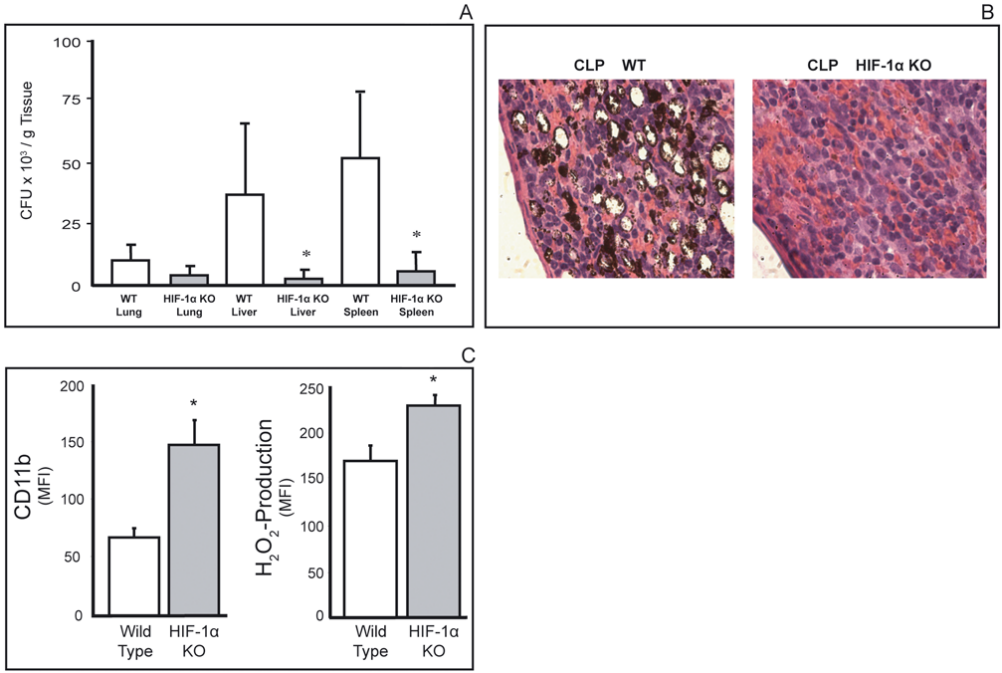
Decreased bacterial burden in mice with T-cell targeted deletion of HIF-1 α. A: *T cell lineage specific HIF-1* α *deficient mice have much less bacterial burden in liver and spleen 72 hrs after CLP* *:p<0.05 vs. WT, means±SEM, N = 3 per group. B: *Growth of gas-forming bacteria and tissue destruction during CLP-induced sepsis in mice with HIF-1* α*-expressing T cells, but not in mice with HIF-1* α *gene–deleted T cells.* Masses of bacteria form rings around gas bubbles in spleens of mice with HIF-1 α expressing T cells. Much less bacteria could be seen in the spleen taken 72 h after CLP from mice with HIF-1 α deficiency in T cells. C: *T-cell specific deficiency in HIF-1 α enhances effector functions of bactericidal granulocytes.* Left Panel: Much stronger upregulation of activation marker CD11b on tissue granulocytes (CD11b^+^/Gr-1^bright^ cells) isolated from HIF-1 α-deleted mice compared to HIF-1 α -expressing control mice. *:p<0.05 vs. WT, N = 3. Right Panel: Higher spontaneous production of hydrogen peroxide by tissue granulocytes (CD11b^+^/Gr-1^bright^ cells) in HIF-1 α-deleted mice than in HIF-1 α-expressing control mice. *:p<0.05 vs. WT, N = 3.

The protective effects of genetic inactivation of HIF-1α in T cells are most likely due to the relief from the negative regulation by HIF-1α of pro-inflammatory, anti-bacterial functions of T cells. This is reflected in the strong attenuation of bacterial burden in spleen and liver of HIF-1 α-deleted mice (**Fig. 2A**). The quantitative data showing that the decrease in bacterial counts are paralleled by a dramatic decrease in the number of gas-forming bacteria in HIF-1 α-deficient mice. For instance, **Fig. 2B** shows that masses of gas-forming bacteria formed rings around gas bubbles that also contained free bacteria in the spleen of HIF-1 α-expressing mice. Such intensive bacterial growth was not observed in littermates with HIF-1 α-deficient T cells. These data provide direct microbiological and histological evidence that HIF-1 α inhibits anti-bacterial activities of T cells (**Fig. 2A,B**). Inactivation of HIF-1 α in T cells might contribute to amplification of bactericidal functions of cells of the innate immune system. Accordingly, granulocytes isolated from septic mice with T cell specific HIF-1 α deficiency were much stronger activated as indicated by levels of CD11b cell surface expression and H_2_O_2_ production (**Fig. 2C**). In agreement with the general view that IFN- γ is a likely candidate for T cell dependent enhancement of bactericidal functions of granulocytes, the observed rescue from septic death and the decrease in the bacteria-mediated tissue damage (**Fig. 1C** **and** **2A,B**) in mice with HIF-1 α protein deficiency in T cells suggested to test for a possible HIF-1α deficiency-induced “de-inhibition” of secretion of pro-inflammatory cytokines in TCR-activated T cells.

### T cell specific deletion of HIF-1 α gene “de-inhibits” TCR-activated T cells and results in increased cell proliferation and cytokines secretion

**Figure 3:**
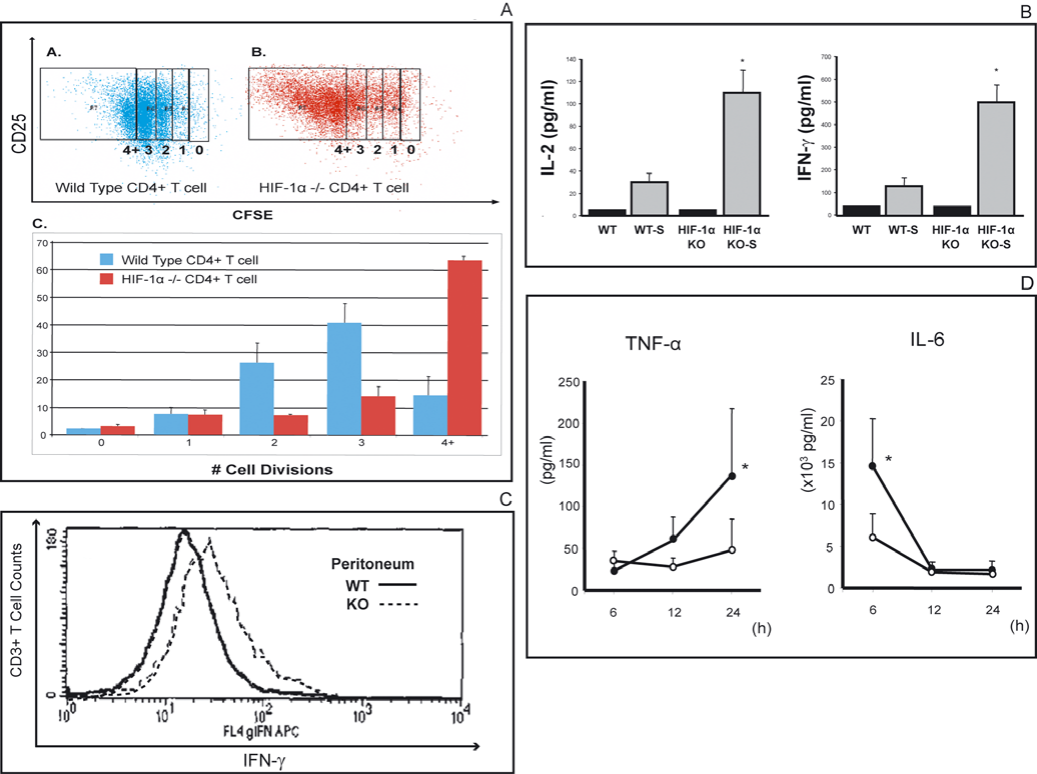
HIF-1α is a negative regulator of TCR-triggered pro-inflammatory cytokine secretion *in vitro* and *in vivo*. A: *T lymphocytes deficient in HIF-1α undergo more cell divisions as compared to wild type T cells.* Splenic T cells were purified, stained using CFSE, activated for 72 hours and analyzed by flow cytometry as described. A representative dot plot of wild type CD4 T cells (A) and HIF-1α deficient T cells (B) showing both activation by CD25 expression and cell divisions. (C) Comparison of number of divisions by CD4 T cells.*: p<0.05 vs. WT, N = 3 per group. B: *T cell specific disruption of HIF-1 α gene substantially increases pro-inflammatory cytokine secretion by ex vivo TCR-activated T cells.* Spleen T cells were derived from T cell lineage specific HIF-1 α deficient mice. Cells were activated for 24 h under hypoxic conditions (1% O_2_). Extracellularly secreted cytokines were determined by ELISA. *:p<0.05 vs. WT, N = 5. C: *Higher intracellular levels of IFN-γ production by inflamed peritoneum-located hypoxic HIF-1 α deficient CD8+ T cells as compared with similarly located in vivo hypoxic HIF-1 α expressing T cells after CLP*. Peritoneal lavage was performed 72 hrs after CLP and 1,5×10^6^ T cells were restimulated and stained with anti-IFN-*γ* mAb. D: *Levels of proinflammatory cytokines TNF-α and IL-6 are significantly higher as compared to mice with selective disruption of HIF-1α gene in T-cells.* Peritoneal fluid (TNF-α, left panel) and serum (IL-6, right panel) were withdrawn at the indicated times after CLP and cytokines were determined by ELISA. Closed circles: HIF-1α KO, open circles: WT. *:p<0.05 vs. WT, N = 4

Genetic deletion of HIF-1α in T cells resulted in increased TCR triggered cell proliferation as shown by significantly higher percentages of cells which had undergone three or four cell divisions upon anti-CD3 mediated activation (**Fig. 3A**). Although enhanced cell proliferation may result from direct relief of cell cycle arrest as induced by loss of HIF-1α e.g. in B cells [11], the increase in IL-2 production we found in HIF-1α deficient T cells might account for enhanced T cell proliferation as well (**Fig. 3B**). Besides IL-2, IFN-γ secretion was significantly increased with the latter confirmed at the intracellular level in HIF-1α deficient peritoneal T cells harvested 72 h after CLP (**Fig. 3C**). No extracellularly secreted IFN-γ could be detected in blood serum or peritoneal lavage fluid neither of HIF-1α KO or HIF-1α WT mice. However, in agreement with IFN-γ acting as an autocrine or paracrine biological immune response modifier which enhances the production of other pro-inflammatory cytokines [12], levels of TNF-α and IL-6 after cecal ligation and puncture were higher in peritoneal lavage fluid and blood serum in HIF-1α KO than in HIF-1α WT mice (**Fig. 3D**)**.**

The here described first evidence of the enhanced *in vivo* pro-inflammatory cytokine production by T cells deleted in HIF-1α by the Cre-lox P system [13] is in agreement with *in vitro* assays of T cell lines independently obtained from chimeric mice, where HIF-1 α was genetically inactivated using RAG-2 gene blastocyst complementation system [14], [15].

### Deletion of HIF-1α enhances TCR stimulated NF-κB activation

**Figure 4:**
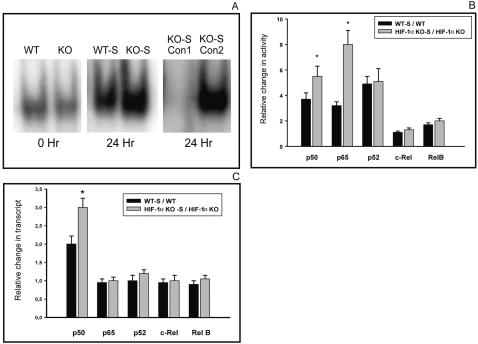
Increased NF-κB m-RNA expression and activity of *ex vivo* activated T cells of mice with T-cell targeted deletion of HIF-1 α. For all three panels, T-cells from spleens were isolated from age and sex matched lck Cre (−) and lck Cre (+) HIF-1α loxP mice and stimulated as described (WT-S, κΟ−S). Unstimulated cells served as controls (WT, κΟ). A: *T cell specific disruption of HIF-1 α gene substantially increases NF-κB binding activity in ex vivo TCR-activated T cells.* Nuclear extracts were prepared from harvested cells and EMSA was conducted. The experiment was repeated and representative data of two experiments are shown. All lanes contain hot binding probe for NF-κB. Specificity of EMSA was tested in the presence of 50 fold excess of either unlabeled probe (Con 1) or CRE specific probe (Con 2), respectively. B: *T cell specific disruption of HIF-1 α gene increases NF-κB p50 and p65 binding activity in ex vivo TCR-activated T cells.* NF-κB-ELISA was conducted with nuclear extracts. *:p<0.05 vs. WT, N = 4. C: *T cell specific disruption of HIF-1 α gene increases NF-κB p50 mRNA expression in ex vivo TCR-activated T cells.* RNA was prepared and subsided to quantitative RT-PCR. *:p<0.01 vs. WT. N = 4.

The NF-κB transcription factors are key regulators of inflammatory and immune response [16]. We hypothesized that observed effects of HIF-1α deletion in T cells may be due to HIF-1α affecting T cells by acting through NF-kB. Indeed, studies of neutrophils did suggest that NF-κB could be hypoxia-regulated and a HIF-dependent target to increase neutrophil survival [17]. Accordingly, we studied the effects of HIF-1α deletion on NF-κB activity in T cells. As shown by EMSA, NF-κB DNA binding activity was enhanced in nuclear extracts of TCR stimulated HIF-1α deficient T cells (**Fig. 4A**). While in neutrophils NF-κB p65 expression plays a major role in HIF-1α dependent regulation of NF-kB activity [17], the TCR-stimulated NF-κB activation in T cells comprises p50+RelA and p50+cRel heterodimers and p50 homodimers [18]. NF-κB family profiling (p65, p50, c-Rel, p52 and RelB ) of DNA binding activity of T cell nuclear extracts showed that upon TCR activation the activity of both the p50 and the p65 subunits significantly increased in HIF-1α deficient cells as compared to T cells with intact HIF-1α genes (**Fig. 4B**). To find out whether these findings are due to NF-κB transcription, we performed quantitative RT-PCR which confirmed a significant increase in NF-κB p50 mRNA expression in HIF-1α deficient T cells (**Fig. 4C**). Thus, HIF-1α is likely to suppress TCR stimulated T cell responses by preventing p50 transcription and thereby inhibiting NF-κB activation. Although HIF-1α is capable to directly inhibit transcription (e.g. CAD gene [19] or ENT gene [20]), we were not able to identify putative HRE sites in the promoter region of the p50 encoding gene [21] by analyses using a hidden Markov model (HMM) algorithm. Alternatively, HIF-1α might affect a multiplicity of yet unknown proteins or transcription factors influencing the p50 transcription and therefore may indirectly suppress p50 expression.

The reported here consideration and studies of an inhibitory role of HIF-1α in regulation of activated T cells have been prompted by several lines of earlier observations: i) hypoxia inhibited the TCR-induced pro-inflammatory cytokine (e.g. IFN- γ) accumulation by T cells [10]; ii) the deletion of HIF-1α in T and B cells resulted in an increased tissue damage and autoimmunity [14] and iii) the deletion of HIF-1α in T cells resulted in enhanced activation of T cells *in vitro* [15].

The data presented here provide the first direct genetic evidence for the negative regulation of T cell activities by HIF-1α *in vivo*. Furthermore, we show that the observed anti-inflammatory effect of HIF-1α during anti-bacterial response results from suppression of T cell proliferation and pro-inflammatory cytokine production most likely due to inhibition of NF-κB/p50 transcription and subsequent reduction of NF-κB activity.

Of potential clinical significance, these observations offer a new approach to enhance the immune response by de-inhibiting T cells from HIF-1α–mediated immunosuppression and thereby enhancing production of IFN-γ by T cells *in vivo*. This may-in turn-lead to a greater activation of cells of the innate immune system, which then play a key role in the early bacterial elimination. Our findings may re-invigorate the attention to the anti-bacterial effects of T cells and appeal to recruit T cells' powerful, but otherwise unavailable anti-bacterial capacity in novel anti-sepsis therapies. Moreover, our observations of T cells inhibition by HIF-1α in hypoxic inflamed peritoneum suggest that activated anti-tumor T cells may be also inhibited near or within hypoxic tumors by HIF-1α. The HIF-1α-mediated inhibition may be additive or synergistic with immunosupression caused by tumor hypoxia-produced extracellular adenosine that is protecting tumors by inhibiting the incoming anti-tumor T cells via their A_2A_ adenosine receptors [22].

## Materials and Methods

### Generation of lck-Cre+/HIF1-α loxP floxed/floxed mice

Mice with T cell-targeted deletion of HIF-1 α gene have been generated as described in [15]. This allows for a T cell lineage specific HIF-1 α deletion in lck-Cre positive mice, while the lck-Cre negative littermates are HIF-1 α positive and can be used as controls. The gross phenotype of the lck-Cre (+) HIF-1 α loxP mouse was not markedly different from lck Cre (−) HIF-1 α loxP mouse (HIF-1 α expressing littermates) as judged by viability, weight, or limb deformities.

### Determination of deletion efficiency

For the measurement of deletion efficiency of HIF-1 α in lck-Cre (+) mice the purified T cells were obtained by positive selection on AutoMACS cell separator using anti-CD3 mAb and HIF-1 α mRNA expression was determined by real time RT-PCR as in [23] using primers spanning the targeted region as well as primers for an undeleted control gene for normalization.

### Cecal ligation and puncture

To enable the investigation of the possible role of T cells in clearing bacterial infections, we have adapted a murine model of sepsis, which results in 50% mortality after 72 hours of cecal ligation and puncture (CLP) [8]. By avoiding the early (<24 hour) lethal events of sepsis enabled us to study the behavior of T cells, which may have required longer than 24 hrs to be recruited and activated in inflamed environment. CLP was performed always by the same scientist who was blinded to the experimental design until the final time point of experiment (death of mice). Sham-treated controls underwent the same surgical procedures (i.e. laparotomy and resuscitation), but the cecum was neither ligated nor punctured.

### Bacterial Forming Colony Analysis

Bacterial counts were performed on aseptically harvested spleens, lungs and livers as described previously [24]. The tissues were weighed, homogenized, and cellular debris pelleted. The supernatant samples were serially diluted in sterile saline and cultured on tryptic soy agar pour plates. Plates were incubated for 48 hours after which colony counts were performed. Data are expressed as colony counts per gram tissue.

### Tissue Damage Assessment

The spleen, liver and lung were harvested from sham and CLP mice and placed into 4% paraformaldehyde. The tissue were sliced and stained according to standard protocols used by American Histolabs, Gaithersburg, MD.

To estimate hepatocellular injury during sepsis, ALT serum activity was determined as previously described [6].

### Analysis of granulocyte functions in extraperitoneal organs

Functions of granulocytes–production of hydrogen peroxide and expression of CD11b–were determined as previously described [2].

### Isolation of mononuclear cells from spleen, lymph nodes and peritoneum

Spleens (lymph nodes) were homogenized using a 70 µm cell strainer. Cells from the peritoneum were collected by insertion and withdrawing of 10 ml of sterile PBS from the peritoneal space. Purified T cells were obtained by negative selection on AutoMACS cell separator using the Pan T-cell isolation kit (Miltenyi Biotech) and placed in RPMI 1640 (Biofluids) supplemented with 5% dialyzed FCS (heat inactivated) and 100 U/ml penicillin, 100 mg/ml streptomycin, 1 mM sodium pyruvate, 1 mM HEPES, and non-essential amino acids (RP5). Viability was assessed using trypan blue.

### Cytokine measurements

Intracellular staining of TCR-activated cells to evaluate IFN-γ production *in situ* was performed in permeabilized cells (after 4 h of anti-CD3 and anti-CD28 mAb restimulation) by flow cytometry analysis using anti-cytokine mAB (BD Pharmingen), 2 µM (final concentration) monensin (Cal-Biochem) and saponin buffer (PBS containing 0,1% (w/v) saponin, 0,1% BSA, 0,01 M HEPES and 0,1% sodium azide) as described [10] Extracellular cytokine concentrations from serum, peritoneal fluid and from cell culture supernatant were determined by ELISA kits (BD Pharmingen) according to the manufacturer's instructions.

### Flow cytometry

Analyses of cell surface antigen expression and of IFN-γ expression *in situ* were performed as described earlier [10]. Flow cytometry data acquisition and analysis were done on LSR, DIVA and CellQuestPro programs (Becton Dickinson). All mAb used for cytometry were obtained from BD Pharmingen.

### *Ex vivo* activation of T cells

Purified T-cells were cultured in 12-well plates pre-coated with anti-CD3 and anti-CD28 mAb 3 micro g/ml each. (Culture volume was 1 ml per well containing 3×10^6^ cells.)

Cells were cultured in normoxic (21% O_2_) or hypoxic (1% O_2_) environments. Normoxia was controlled by using a humidified 5% CO_2_/air incubator, and hypoxia by pregasing culture medium for 10 min in a sealed hypoxic work station with 5% CO_2_/balance N_2 _gas mix and subsequent culture in a humidified hypoxic (CO_2_/N_2_) incubator. Supernatants and cells were harvested 24 h later.

### Analysis of cell proliferation

Purified splenic T cells were stained using 5,6-carboxyfluorescein diacetate succinimidyl ester (CFSE), activated by platebound 3 micro g/mL anti-CD3 and anti-CD28 mAb for 72 hours and analyzed by flow cytometry as described [25].

### NF-κB DNA binding activity

Nuclear proteins were extracted from 1,5×10^7^ T-cells using the Active Motif Nuclear Extract kit according to the manufacturer's instructions, total protein concentrations of the lysates were determined by Bradford assay (Bio-Rad). EMSA was performed as in [26]. The DNA binding activity of the NF-κB subunits in 5 µg of T-cell nuclear extracts was quantified by an ELISA assay using the trans-AM NF-κB family transcription factor assay kit (Active Motif North), according to the manufacturer's protocol.

### Quantitative RT-PCR

Spleen samples were homogenized before Total RNA isolation using the RNeasy mini kit (Qiagen) including DNAse treatment following the manufacturer's protocol. Equal amounts of RNA from the different samples were transcribed into cDNA using the Superscript first strand RT-PCR kit (Invitrogen). Real-time qPCR was performed in duplicates with the Light Cycler480 instrument (Roche Diagnostics) using the Roche's SYBR-Green mastermix and prevalidated primer pairs (Qiagen). The thermal cycler conditions were 35 cycles of 94°C for 20 s, 55°C for 20 s, and 72°C for 30 s. Relative mRNA expression was calculated with the Relative Quantification software (Roche Diagnostics) using an efficiency-corrected algorithm with standard curves and 18S ribosomal RNA as an endogenous reference.

### Statistical Analysis

All data were analyzed using GraphPad Prism software (GraphPad Software). If otherwise not stated, values given represent means±SD. Intergroup comparisons were performed by unpaired T-Test or Mann-Whitney Test, if data were normal or not normally distributed, respectively. Survival curves were compared using the Log Rank test. Two-tailed levels of statistical significance are indicated by *:p<0.05.
